# Evaluating the Quantitative Foveal Avascular Zone and Retino-Choroidal Vessel Density Using Optical Coherence Tomography Angiography in a Healthy Indian Population

**DOI:** 10.7759/cureus.27669

**Published:** 2022-08-04

**Authors:** Punita K Sodhi, Ekta Shaw, Akanksha Gautam, Alka Yadav, Archana T R, Kavya C Rao, Shantanu Sharma, Ruchir Tewari

**Affiliations:** 1 Ophthalmology, Guru Nanak Eye Centre and Maulana Azad Medical College, New Delhi, IND; 2 Preventive and Social Medicine, Mamta Health Institute for Mother and Child, New Delhi, IND; 3 Ophthalmology, Tewari Eye Centre, Noida, IND

**Keywords:** indian population, choroid, retina, vessel density, foveal avascular zone, optical coherence tomography angiography

## Abstract

Objective: The foveal avascular zone (FAZ) is the round capillary-free zone within the macula and is supplied only by a single-layered parafoveal capillary arcade. This study aimed to evaluate the quantitative FAZ and retino-choroidal vessel density (VD) using optical coherence tomography angiography (OCTA) in a healthy Indian population.

Methods: This was a cross-sectional observational study that was conducted for evaluating the quantitative FAZ and retino-choroidal VD of 200 eyes of 100 healthy Indian subjects, including 62 males and 38 females (age range 17-50 years) having the best-corrected visual acuity (BCVA) of logMAR 0 (20/20; 6/6) and spherical equivalent refractive error of not more than 1 D. The subjects were examined using OCTA automated software on spectral-domain OCT (SD-OCT; Nidek RS 3000 Advance 2; Nidek, Inc., Fremont, CA) on a 3 x 3 mm OCTA macular scan centred on the fovea. The FAZ size, perimeter and circularity index, VD in superficial, deep, and outer retina (OR), outer retinal chorio-capillaries (ORCC), chorio-capillaries (CC) and choroid (C) were analysed in the circular and quadrant-segmented zones. A correlation was found between the FAZ size, perimeter and circularity, and VD in retino-choroidal layers, and between BCVA, age, central foveal thickness (CFT) and sub-foveal choroidal thickness (SFCT), and OCTA parameters.

Results: The FAZ and surrounding vascular arcades were intact in all eyes, showing either a vertical or horizontal oval-shaped symmetrical formation without gaps, holes or interruption of the capillary network. The mean value of CFT was 237.5±26.0 microns and SFCT was 269.6±53.0 microns. The mean FAZ area was 0.42±0.23 mm^2^, FAZ perimeter was 3.3±1.0 mm and FAZ circularity index was 0.46±0.1. The mean VD in superficial capillary plexus (SCP) was 23.87±10.66, in deep capillary plexus (DCP) was 16.03±9.90, in OR was 13.22± 12.27, in ORCC was 39.74±14.32, in CC was 37.02±16.43 and in choroid was 37.43±16.76. The increasing order of VD in different retino-choroidal layers was OR<DCP<SCP<CC<C<ORCC. The FAZ area and perimeter had a statistically significant (SS) negative correlation with the VD in SCP, DCP and OR, while the FAZ perimeter additionally had a SS negative correlation with the VD in the choroid. The FAZ circularity had a SS positive correlation with VD in SCP, DCP and OR. There was no SS correlation between the age of subjects and OCTA parameters. The BCVA had a SS correlation with only one parameter, i.e. ORCC foveal (r=-0.15; p=0.03). There was a SS negative correlation between CFT and FAZ area and perimeter, and a SS positive correlation between the CFT and VD in SCP, DCP and OR. There was a SS positive correlation between the SFCT and VD in different layers including SCP, DCP, ORCC, CC and choroid.

Conclusions: VD is the highest in the ORCC and lowest in the OR segment. A better VD in SCP, DCP and OR is associated with a reduced FAZ area/FAZ perimeter and an increased FAZ circularity index. A lower VD in the ORCC is associated with worse BCVA. In a healthy subject, a relatively thicker foveal region is likely to have better vascularity of SCP, DCP and OR and a smaller FAZ area/FAZ perimeter. In the absence of diseases, relatively thicker sub-foveal choroid is expected to have better vascularity of SCP, DCP, ORCC, CC and choroid.

## Introduction

Physiologically, the retina, especially the macula, requires more oxygen per weight than any other tissue, and thus, is more prone to hypoxic damage [1]. As 50% retinal ganglion cells are distributed in the macula, examining macular circulation is very vital [2]. The pachychoroid spectrum of diseases is claimed to lead to an increased choroidal blood flow, and this can be quantified through the measurement of choroidal vessel density (VD).

The foveal avascular zone (FAZ) is the round capillary-free zone within the macula and is supplied only by a single-layered parafoveal capillary arcade. Thus, the FAZ area reflects vascular compromise early in the course of disease. The FAZ circularity index quantifies the breakdown of the parafoveal capillary network, and thus may be the more sensitive indicator for detecting an early vascular damage [3,4]. A subtle change in the parafoveal vascular arcade because of age, diabetic retinopathy and retinal vascular occlusive diseases may reduce the FAZ circularity index even before an enlargement of the FAZ area in advanced disease [5,6]. The FAZ parameters correlate significantly with macular ganglion cells and inner plexiform layer thickness, peripapillary retinal nerve fibre layer (RNFL) thickness and central visual field defects [3,7]. A multi-layered vascular plexus supplies macula outside fovea, and hence, it is crucial to study the sectoral macular VD adjoining fovea [3].

Data on FAZ parameters including size, perimeter and circularity, and VD of different retinal and choroidal layers is not available for the healthy Indian population as seen in a PubMed search in different languages. Additionally, the visualization of VD in chorio-capillaries and choroid is a relatively unexplored area. We examined healthy Indian subjects with 3 x 3 mm optical coherence tomography angiography (OCTA) scans centered on fovea using spectral-domain optical coherence tomography (SD-OCT) to see the FAZ size, perimeter and circularity index, and VD in the superficial, deep, and outer retina, outer retina chorio-capillaries, chorio-capillaries and choroid.

This study was originally presented as a poster at the 80th All India Ophthalmological Conference 2022, Mumbai, India, on June 03, 2022.

## Materials and methods

This cross-sectional observational study conformed to the tenets of the Declaration of Helsinki and was approved by the institutional ethical committee (approval F 1/IEC/MAMC/70/05/2019/No.506) of the Maulana Azad Medical College, New Delhi; the study included a control group of subjects during the one-year period of 2019-2020. Following informed consent, 100 healthy Indian subjects, including 62 males and 38 females, within the age range of 17 to 50 years were enrolled in this study. The study was performed at the Department of Ophthalmology, Guru Nanak Eye Centre and Maulana Azad Medical College, New Delhi.

Each subject underwent ophthalmic examination for best-corrected visual acuity (BCVA) on the ETDRS distance chart, manifest refraction, slit-lamp biomicroscopy, Goldman applanation tonometry, and dilated stereoscopic examination of the optic disc and retina. Only healthy individuals having normal fundus on clinical funduscopy and spectral-domain optical coherence tomography (SD-OCT) imaging in both eyes were included in this study. The subjects had the BCVA of logMAR 0 (20/20; 6/6), spherical equivalent refractive error of not more than 1 D, axial length 22-24 mm and intraocular pressure less than or equal to 21 mmHg [8]. They had clear media ensuring good quality scans of the macula. They did not have any significant ocular affection like glaucoma, ocular hypertension and optic neuropathy; coexisting retinal diseases including retinal vaso-occlusive diseases; systemic disease like diabetes mellitus, hypertension and cardiovascular disease; neurological diseases or prior surgery or laser treatment.

The subjects were examined using OCTA automated software on SD-OCT (Nidek RS 3000 Advance 2; Nidek, Inc., Fremont, CA) on a 3 x 3 mm volumetric scan of the macula centered on the fovea. Imaging was performed with pupil dilation. The device obtains 85,000 A-scans per second using a light source at 880 nm. A standardized imaging protocol was used to obtain the OCT scans performed on one eye by a trained operator under standardized mesopic light conditions. The equipment uses real-time compensation for eye movement with a scanning laser ophthalmoscope-based eye tracker resulting in more accurate scans, ensuring a higher image quality and maximum reproducibility. The built-in segmentation algorithm automatically detects the boundaries of the retinal and choroidal layers from the structural OCT cross-sectional images and the en face OCTA scan is then produced by decorrelation projection within the segmented slab. The images acquired include superficial capillary plexus (SCP), deep capillary plexus (DCP), outer retinal (OR) plexus, outer retinal chorio-capillaries (ORCC), chorio-capillaries (CC) and choroidal (C) plexus for VD.

The measurements of VD in OCTA was done in three circles centred on the fovea, including an inner circle of 0.5 mm diameter, i.e. foveola; middle circle having inner and outer ring diameters of 0.5 and 1.5 mm, respectively, i.e. parafovea and outer circle having inner and outer ring diameters of 1.5 and 3 mm, respectively, i.e. perifovea. VD was reported as the percentage of the total area occupied by large vessels and microvasculature blood within these regions [9].

FAZ is defined as the area encompassing the central fovea without any vessels and the area is delineated by the equipment along the centremost capillaries to enable the calculation of the FAZ area and perimeter. The central foveal thickness (CFT) is the retinal thickness within the central ring having a diameter of 1 mm. The circularity index is a measure of maintenance of a round shape and it is calculated as function of the perimeter. The circularity index of a perfect circle is 1.0. Thus, a ratio closer to 0 indicates the most irregular shape and disruption of the parafoveal capillary network, while that closer to 1.0 indicates similarity to a perfect circle and intactness of the parafoveal network [9].

The scans having projection or motion artifacts like vessel doubling or edge duplication were excluded. All images were taken at the signal strength index (SSI) of 90%. The scans were taken three times and readings were averaged. We examined OCTA scan images at the site of acquisition and stored these by exporting images where these could be independently assessed later using Image J (version 1.49; National Institutes of Health, Bethesda, MD). The software automatically calculated size, perimeter and circularity index of FAZ. In addition to the foveal VD, the parafoveal VD and perifoveal VD in different layers were analysed in the circular and 90° quadrant-segmented zones. The quadrants were temporal, superior, nasal and inferior.

The SCP was bracketed between internal limiting membrane (ILM) and inner plexiform layer/inner nuclear layer (IPL/INL), i.e. between ILM till 13 microns below IPL/INL; the DCP was bracketed between IPL/INL and outer plexiform layer/outer nuclear layer (OPL/ONL), i.e. at a depth starting at 8 microns below IPL/INL till 13 microns below OPL/ONL; the OR was bracketed between OPL/ONL and retinal pigment epithelium/Bruch’s membrane (RPE/BM), i.e. at a depth starting at 8 microns below OPL/ONL till 71 microns above RPE/BM. The ORCC was bracketed between OPL/ONL and RPE/BM, i.e. at a depth starting at 8 microns below OPL/ONL till 21 microns below RPE/BM. The chorio-capillaries was bracketed at the level of RPE/BM, i.e. at a depth starting at 4 microns below RPE/BM till 32 microns below RPE/BM). The choroid was bracketed at the level of RPE/BM, i.e. at a depth starting at 25 microns below RPE/BM till 63 microns below RPE/BM [10]. Quantitative measurements of the retino-choroidal VD were analysed using en face projection images.

The OCT scans were examined to look for abnormalities like exudates, edema, neuro-sensory detachment, pigment epithelial detachment and epiretinal membranes. We characterized the fovea in SD-OCT as characteristic foveal depression where there is a lack of retinal layers including nerve fibre layer, ganglion cell layer, inner nuclear layer and inner plexiform layer [11]. CFT was measured as the distance between the hyperreflective line corresponding to the RPE and ILM [11]. Sub-foveal choroidal thickness (SFCT) was defined as a perpendicular distance between BM and the choroidal scleral junction measured at the centre of the fovea [10].

The primary outcome parameter was OCTA parameters and the secondary outcome parameter was OCT parameters.

Statistical analysis

Statistical analysis was performed with SPSS, version 20.0 for Windows (IBM Corp., Armonk, NY). Descriptive statistics for demographic, ocular, OCT and OCTA parameters were calculated as means ± standard deviations. The normality of the data distribution was tested using the Kolmogorov-Smirnov test. For non-parametric data, median values and interquartile range (IQR) were also calculated. A sample size of 200 eyes of 100 healthy subjects, keeping 10% margin of uncertainty, was used to find correlations in the present study. A correlation between FAZ-related parameters (size, perimeter and circularity) and values of VD in retino-choroidal layers, and a correlation between BCVA, age, CFT and SFCT, and OCTA parameters (including FAZ-related parameters and VD in retino-choroidal layers) were made using Spearman’s correlation coefficient. A p-value of <0.05 was taken as statistically significant (SS).

## Results

This study included 200 eyes of 100 healthy volunteers (62 males and 38 females). The mean age of participants was 32.92±9.14 years, and the mean axial length was 22.76±4.04 mm. The OCT scans were normal in all subjects. The FAZ and surrounding vascular arcades, a circular system of capillaries, were intact in all eyes, showing either a vertical or horizontal oval-shaped symmetrical formation without gaps, holes or interruption of the capillary network. Table 1 presents the demographic, ocular, OCT and OCTA parameters including CFT, SFCT, FAZ area, perimeters and circularity and VD in foveal, parafoveal and perifoveal retinal and choroidal layers.

**Table 1:** Demographic, ocular, OCT and OCTA parameters of healthy subjects IQR: interquartile range; CFT: central foveal thickness; SFCT: sub-foveal choroidal thickness; FAZ: foveal avascular zone; VD: vessel density; SCP: superficial capillary plexus; DCP: deep capillary plexus; OR: outer retina; ORCC: outer retinal chorio-capillaries; CC: chorio-capillaries; OCT: optical coherence tomography; OCTA: optical coherence tomography angiography *Non-parametric data

S. no.	Parameters	Mean±SD (range)	Median and IQR (for non-parametric data)
1.	Age (years)	32.9±9.1 (17 to 50)	-
2.	Intraocular pressure (mmHg)	17.0±1.8 (14 to 20)	-
3.	Axial length (mm)	22.1±3.4 (21 to 24)	-
4.	Refractive error (in spherical equivalent dioptres)*	0.08±0.74 (-1 to +1)	0.5 (-0.5 to 0.75)
5.	CFT (microns)	237.5±26.0 (184 to 340)	-
6.	SFCT (microns)	269.6±53.0 (118 to 402)	-
7.	FAZ area (mm^2^)*	0.42±0.23 (0.02 to 2.04)	0.38 (0.30-0.47)
8.	FAZ perimeter (mm)	3.3±1.0 (0.68 to 7.71)	-
9.	FAZ circularity index	0.46±0.1 (0.21 to 0.71)	-
10.	VD SCP foveal*	5.20±7.19 (0 to 52)	3.0 (1.0-7.0)
11.	VD SCP parafoveal*	26.61±13.42 (1 to 50.5)	29.7 (14.0-38.2)
12.	VD SCP perifoveal*	39.81±15.83 (6 to 56)	48.5 (21.0-53.7)
13.	VD SCP whole*	23.87±10.66 (2.33 to 47.33)	27.3 (12.4-32.5)
14.	VD DCP foveal*	2.71±5.68 (0 to 48)	0 (0-3)
15.	VD DCP parafoveal*	15.79 ±11.34 (0 to 49)	13.0 (7.2-22.1)
16.	VD DCP perifoveal*	29.59±16.45 (0 to 55.25)	27.0 (16.2-46.1)
17.	VD DCP whole*	16.03±9.90 (0 to 40.33)	13.9 (9.0-23.7)
18.	VD OR foveal*	13.58±14.79 (0 to 59)	8.0 (2.0-19.0)
19.	VD OR parafoveal*	16.89±16.05 (0 to 64.75)	11.5 (4.8-25.7)
20.	VD OR perifoveal*	9.20±8.71 (0 to 34.75)	6.0 (2.7-13.0)
21.	VD OR whole*	13.22±12.27 (0 to 48.58)	8.6 (3.3-20.5)
22.	VD ORCC foveal*	37.75±17.54 (0 to 69)	40.0 (22.2-53.7)
23.	VD ORCC parafoveal*	39.71±14.41 (0 to 62.75)	46.0(22.8-50.6)
24.	VD ORCC perifoveal*	41.76±14.04 (9 to 59)	50 (24.0-53.1)
25.	VD ORCC whole*	39.74±14.32 (6 to 60.33)	45.6 (23.2-52.0)
26.	VD CC foveal*	37.15±18.42 (0 to 69)	40.0 (20.0-54.0)
27.	VD CC parafoveal*	37.02±16.85 (0 to 65)	41.2 (20.2-52.0)
28.	VD CC perifoveal*	36.88±16.12 (3 to 57)	44.1 (20.0-51.5)
29.	VD CC whole*	37.02±16.43 (2.67 to 61.58)	43.0 (20.0-51.9)
30.	VD choroid foveal*	38.10±18.48 (0 to 66)	45.0 (17.0-54.0)
31.	VD choroid parafoveal*	36.92±16.62 (0 to 60)	45.8 (17.0-50.1)
32.	VD choroid perifoveal*	37.27±16.92 (2 to 56.25)	47.8 (16.8-51.2)
33.	VD choroid whole*	37.43±16.76 (1 to 59.33)	45.0 (17.1-51.6)

The mean value of CFT was 237.5±26.0 microns, SFCT was 269.6±53.0 microns, FAZ area was 0.42±0.23 mm^2^, FAZ perimeter was 3.3±1.0 mm and FAZ circularity index was 0.46±0.1; VD in SCP was 23.87±10.66, in DCP was 16.03±9.90, in OR was 13.22±12.27, in ORCC was 39.74±14.32, in CC was 37.02±16.43 and in choroid was 37.43±16.76. Table 2 shows the correlation between FAZ-related parameters and VD in different retino-choroidal layers.

**Table 2:** Correlation between FAZ-related parameters and VD in different retino-choroidal layers FAZ: foveal avascular zone; VD: vessel density; SCP: superficial capillary plexus; DCP: deep capillary plexus; OR: outer retina; ORCC: outer retinal chorio-capillaries; CC: chorio-capillaries *Spearman’s correlation coefficient **Statistically not significant correlation

S. no.	Parameters	FAZ area, r^*^ (p-value)	FAZ perimeter, r^*^ (p-value)	FAZ circularity, r^*^ (p-value)
1.	VD SCP foveal	-0.30 (<0.001)	-0.27 (<0.001)	0.02 (0.73)^**^
2.	VD SCP parafoveal	-0.31 (<0.001)	-0.39 (<0.001)	0.20 (0.003)
3.	VD SCP perifoveal	-0.08 (0.22)^**^	-0.19 (0.003)	0.14 (0.04)
4.	VD SCP whole	-0.28 (<0.001)	-0.35 (<0.001)	0.14 (0.03)
5.	VD DCP foveal	-0.41 (<0.001)	-0.37 (<0.001)	0.009 (0.90)^**^
6.	VD DCP parafoveal	-0.30 (<0.001)	-0.41 (<0.001)	0.29 (<0.001)
7.	VD DCP perifoveal	-0.15 (0.03)	-0.29 (<0.001)	0.29 (<0.001)
8.	VD DCP whole	-0.25 (<0.001)	-0.36 (<0.001)	0.28 (<0.001)
9.	VD OR foveal	-0.14 (0.03)	-0.26 (<0.001)	0.22 (<0.001)
10.	VD OR parafoveal	-0.13 (0.05)	-0.27 (<0.001)	0.26 (<0.001)
11.	VD OR perifoveal	-0.07 (0.29)^**^	-0.20 (0.004)	0.22 (0.002)
12.	VD OR whole	-0.13 (0.06)	-0.26 (<0.001)	0.24 (<0.001)
13.	VD ORCC foveal	-0.004 (0.95)^**^	-0.06 (0.39)^**^	0.003 (0.96)^**^
14.	VD ORCC parafoveal	-0.08 (0.23)^**^	-0.13 (0.05)	0.03 (0.67)^**^
15.	VD ORCC perifoveal	-0.06 (0.37)^**^	-0.10 (0.15)^**^	0.02 (0.71)^**^
16.	VD ORCC whole	-0.05 (0.46)^**^	-0.11 (0.12)^**^	0.03 (0.66)^**^
17.	VD CC foveal	-0.06 (0.33)^**^	-0.07 (0.26)^**^	-0.07 (0.32)^**^
18.	VD CC parafoveal	-0.08 (0.21)^**^	-0.13 (0.05)	0.02 (0.69)^**^
19.	VD CC perifoveal	-0.08 (0.24)^**^	-0.16 (0.01)	0.10 (0.16)^**^
20.	VD CC whole	-0.08 (0.24)^**^	-0.12 (0.08)^**^	-0.01 (0.88)^**^
21.	VD choroid foveal	-0.06 (0.33)^**^	-0.14 (0.03)	0.06 (0.36)^**^
22.	VD choroid parafoveal	-0.09 (0.16)^**^	-0.14 (0.04)	0.04 (0.52)^**^
23.	VD choroid perifoveal	-0.12 (0.09)^**^	-0.15 (0.03)	0.03 (0.63)^**^
24.	VD choroid whole	-0.10 (0.15)^**^	-0.16 (0.02)	0.05 (0.44)^**^

The FAZ area and perimeter had a SS negative correlation with the VD in the SCP, DCP and OR, while the FAZ perimeter additionally had a SS negative correlation with VD in the choroid. The FAZ circularity had a SS positive correlation with VD in the SCP, DCP and OR. Table 3 shows a correlation between OCTA parameters with BCVA, age, CFT and SFCT.

**Table 3:** Correlation of OCTA parameters with BCVA, age, CFT and SFCT BCVA: best-corrected visual acuity; CFT: central foveal thickness; SFCT: sub-foveal choroidal thickness; FAZ: foveal avascular zone; VD: vessel density; SCP: superficial capillary plexus; DCP: deep capillary plexus; OR: outer retina; ORCC: outer retinal chorio-capillaries; CC: chorio-capillaries; OCTA: optical coherence tomography angiography *Spearman’s correlation coefficient **Statistically significant correlation

S. no.	OCTA parameters	BCVA, r^*^ (p-value)	Age, r^*^ (p-value)	CFT, r^*^ (p-value)	SFCT, r^*^ (p-value)
1.	FAZ area (mm^2^)	-0.069 (0.33)	-0.03 (0.61)	-0.37 (<0.001)^**^	-0.01 (0.87)
2.	FAZ perimeter (mm)	-0.072 (0.31)	-0.002 (0.98)	-0.37 (<0.001)^**^	-0.03 (0.61)
3.	FAZ circularity	0.076 (0.28)	-0.012 (0.86)	0.11 (0.10)	0.07 (0.31)
4.	VD SCP foveal	0.069 (0.33)	-0.01 (0.80)	0.211 (0.003)^**^	0.10 (0.13)
5.	VD SCP parafoveal	0.012 (0.86)	0.03 (0.65)	0.179 (0.01)^**^	0.24 (<0.001)^**^
6.	VD SCP perifoveal	-0.013 (0.86)	0.003 (0.97)	0.02 (0.69)	0.25 (<0.001)^**^
7.	VD SCP whole	0.009 (0.89)	0.02 (0.75)	0.158 (0.02)^**^	0.25 (<0.001)^**^
8.	VD DCP foveal	-0.05 (0.48)	0.09 (0.18)	0.21 (0.003)^**^	-0.01 (0.79)
9.	VD DCP parafoveal	-0.017 (0.81)	0.02 (0.69)	0.16 (0.02)^**^	0.15 (0.03)^**^
10.	VD DCP perifoveal	0.009 (0.90)	0.001 (0.98)	0.09 (0.19)	0.22 (0.001)^**^
11.	VD DCP whole	-0.001 (0.99)	0.02 (0.72)	0.11 (0.09)	0.20 (0.004)^**^
12.	VD OR foveal	0.11 (0.10)	0.09 (0.18)	0.16 (0.02)^**^	0.05 (0.43)
13.	VD OR parafoveal	0.07 (0.31)	0.07 (0.30)	0.13 (0.06)	0.06 (0.35)
14.	VD OR perifoveal	0.07 (0.32)	0.08 (0.21)	0.09 (0.19)	0.07 (0.29)
15.	VD OR whole	0.07 (0.27)	0.09 (0.18)	0.14 (0.04)^**^	0.07 (0.30)
16.	VD ORCC foveal	-0.15 (0.03)^**^	-0.002 (0.98)	-0.02 (0.77)	0.24 (<0.001)^**^
17.	VD ORCC parafoveal	-0.122 (0.08)	0.02 (0.91)	0.009 (0.90)	0.23 (0.001)^**^
18.	VD ORCC perifoveal	-0.01 (0.88)	-0.03 (0.58)	-0.02 (0.71)	0.17 (0.01)^**^
19.	VD ORCC whole	-0.122 (0.08)	-0.007 (0.92)	-0.01 (0.82)	0.24 (<0.001)^**^
20.	VD CC foveal	-0.09 (0.17)	0.03 (0.64)	0.007 (0.92)	0.21 (0.002)^**^
21.	VD CC parafoveal	-0.08 (0.24)	0.02 (0.77)	0.02 (0.73)	0.22 (0.002)^**^
22.	VD CC perifoveal	-0.07 (0.27)	0.01 (0.85)	-0.01 (0.78)	0.19 (0.006)^**^
23.	VD CC whole	-0.08 (0.23)	0.02 (0.75)	-0.007 (0.91)	0.23 (0.001)^**^
24.	VD choroid foveal	-0.09 (0.19)	-0.04 (0.54)	0.03 (0.63)	0.21 (0.002)^**^
25.	VD choroid parafoveal	-0.03 (0.60)	-0.005 (0.94)	0.03 (0.63)	0.25 (<0.001)^**^
26.	VD choroid perifoveal	-0.11 (0.09)	0.01 (0.86)	0.003 (0.97)	0.19 (0.006)^**^
27.	VD choroid whole	-0.08 (0.23)	-0.01 (0.82)	0.03 (0.58)	0.22 (0.001)^**^

There was no statistically significant correlation between age of subjects and OCTA parameters. The BCVA had a SS correlation with only one parameter, i.e. ORCC foveal (r=-0.15; p=0.03). The SS negative correlation between the BCVA and ORCC foveal implied that the lesser the VD in ORCC, the more the value of logMAR BCVA, i.e., worse would be the BCVA. There was a SS negative correlation between the CFT and FAZ area and FAZ perimeter, and a SS positive correlation between the CFT and VD in SCP, DCP and OR. There was a negative correlation between the SFCT and FAZ area and FAZ perimeter, though it was not SS. Except for OR, there was a SS positive correlation between the SFCT and VD in different layers including SCP, DCP, ORCC, CC and choroid.

## Discussion

The techniques previously used to visualise the retino-choroidal vasculature including fluorescein angiography and indocyanine angiography have shortcomings like allergic reactions, invasive procedures, contraindications in liver and kidney diseases and most importantly lack of segmentation of retinal and choroidal layers. Other modalities to measure ocular blood flow including color Doppler imaging, laser Doppler flowmetry, retinal vessel analyser and laser speckle flowgraph provide only a partial description of the ocular circulation [3,12]. A qualitative grading of FAZ has shortcomings, such as an absence of a completely standardised method of demarcating fundus landmarks. As there is an overlap of size between healthy subjects and those with abnormalities, it becomes difficult to opine whether a given FAZ is ‘normal’ or ‘pathologic’. Hence, there is a requirement of a quantitative examination technique. Optical coherence tomography angiography is a non-invasive, quick and reliable technique to visualise different layers of the retina and choroid and measure their VD in a quantitative manner.

The superficial capillary plexus and the deep capillary plexus compose the internal blood-retinal barrier [13]. The vascular plexus in chorio-capillaries and larger vessels of the choroid have a significant role in supplying blood to the RPE and OR. This plexus is associated with physiologic changes seen with the increasing age, and retinal diseases like age-related macular degeneration and central serous chorioretinopathy [14]. Indocyanine green angiography lacks depth selectivity and there is difficulty in perception of its emitted cyanescence behind the light-absorbing RPE. Thus, OCTA-based metrics to study capillary networks in the choroid and chorio-capillaries are important biomarkers for retino-choroidal diseases to establish a diagnosis and prognosis [3,7].

The OCTA macular VD parameters are reduced in primary open-angle glaucoma and high myopia [10,15]. But OCTA parameters for VD in retino-choroidal layers have been reported very infrequently. While Abrishami et al., Savastano et al., İçel et al. and Al-Sheikh et al. used 3 x 3 mm scans, Turker et al., Savastano et al., Zhang et al. and González-Zamora et al. used 6 x 6 mm scans and Maruko et al. used 12 x 12 mm scans [10,16-22].

The increasing order of VD in different retino-choroidal layers of our subjects was OR<DCP<SCP<CC<C<ORCC. Our study results showed that the superficial macular VD was greater than the deep macular VD in all analysed regions, similar to the findings of Choi et al. [3]. Compared to other studies, our subjects had a larger FAZ area and FAZ perimeter, lower VD values for SCP and DCP, but higher VD values for the chorio-capillary and choroid layer (Table 4). The subjects from the studies of both Al-Shiekh and Maruko et al. fell in a higher age group, yet SFCT of subjects studied by Al-Sheikh (246.97±41.74 microns) was lesser and that from the study by Maruko et al. (297±61 microns) was more than that of our subjects (269.6±53.0 microns) [10,22]. Even in the thinner choroid, the VD was more in our subjects than that seen by Maruko et al. [22].

**Table 4:** Values for OCTA parameters in healthy population groups OCTA: optical coherence tomography angiography; FAZ: foveal avascular zone; VD: vessel density; SCP: superficial capillary plexus; DCP: deep capillary plexus; CC: chorio-capillaries; C: choroid ^a^Swept source optical coherence tomography ^b^Parafoveal

	Mean age (years)	Authors	Values
FAZ area (mm^2^)	37.44±10.04	Turker et al. [16]	0.29±0.11
	36.6±7.1	Abrishami et al. [17]	0.24±0.08
	44.7±11	Savastano et al. [19]	0.235±0.09
	11.27±3	İçel et al. [20]	0.3±0.09
	5-18	Zhang et al. [18]	0.28±0.10
	32.92±9.14	Our study	0.42±0.23
FAZ perimeter (mm)	44.7±11	Savastano et al. [19]	2.06±0.36
	32.92±9.14	Our study	3.3±1.0
SCP VD	37.44±10.04	Turker et al. [16]	50.53
	60.03±2.33	González-Zamora et al. [21]^a^	29.1±1.88
	36.6±7.1	Abrishami et al. [17]	48.36±2.24
	44.7±11	Savastano et al. [19]	21.20±1.4
	11.27±3	İçel et al. [20]	43.88±3.4
	5-18	Zhang et al. [18]^b^	50.25±4.41
	56.05±19.2	Al-Sheikh et al. [10]	0.325±0.028
	32.92±9.14	Our study	23.87±10.66
DCP VD	37.44±10.04	Turker et al. [16]	54.86
	60.03±2.33	González-Zamora et al. [21]^a^	19.77±1.53
	36.6±7.1	Abrishami et al. [17]	53.03±3.29
	44.7±11	Savastano et al. [19]	23.05±3.44
	11.27±3	İçel et al. [20]	39.6±3.55
	5-18	Zhang et al. [18]^b^	53.9±4.71
	56.05±19.2	Al-Sheikh et al. [10]	0.366±0.032
	32.92±9.14	Our study	16.03±9.90
CC VD	37.44±10.04	Turker et al. [16]	2.08±0.11
	60.03±2.33	González-Zamora et al. [21]^a^	5.03±1.03
	32.92±9.14	Our study	37.02±16.43
C VD	38.5±8.0	Maruko et al. [22]^a^	27.3±8.2
	32.92±9.14	Our study	37.43±16.76

İçel et al. did not find any SS relation between OCTA parameters and gender, refractive error and axial length [20]. Abay et al. studied 153 eyes of 153 healthy individuals aged between 20 and 80 years in 6 x 6 mm retinal OCTA images for SCP VD, DCP VD, FAZ area and CC flow area and compared these values among five age groups. With the increase in age, the VD in the SCP and DCP and CC flow area significantly reduced but the FAZ area increased though this increase was not statistically significant (p=0.660) [23].

We studied a healthy Indian individuals who were emmetropic or had spherical equivalent refractive error of not more than 1 D. Similar to İçel et al., VD in the SCP and DCP had a negative correlation with the FAZ area implying that vascular compromise in these layers would cause a larger FAZ [20]. A similar pattern was observed by us for the FAZ perimeter that had a negative correlation with VD in the SCP, DCP, OR and choroid. The FAZ circularity increased with an increase in VD in the SCP, DCP and OR. There was no significant correlation between age and any OCTA parameter. While CFT had a negative correlation with the FAZ area and perimeter, there was a positive but not significant correlation with the FAZ circularity. This means that a thinner fovea may have a compromised vascularity leading to an increase in the FAZ area and FAZ perimeter and reduction in circularity index. The CFT had a positive correlation with VD in the SCP, DCP and OR; thus, in a healthy individual, thicker fovea is associated with more blood supply in the SCP, DCP and OR. Even the SFCT had a significant positive correlation with VD in all the layers except OR, implying that thicker choroid is accompanied with a better blood supply in a healthy subject. As in our study, Maruko et al. also found a significant positive correlation between SFCT and VD in the choroid (r=0.22, p=0.001) [22]. The logMAR values of BCVA had a significant negative correlation with VD in the ORCC foveal, implying that an improvement in VD in ORCC leads to a reduction in logMAR BCVA or an improved BCVA (Table 5). We did not study the correlation between OCTA parameters and refractive error or axial length as the range for these was very narrow in our subjects. We did not study a variation of OCTA parameters with gender.

**Table 5:** Relation of different OCTA parameters with ocular and demographic features OCTA: optical coherence tomography angiography; FAZ: foveal avascular zone; VD: vessel density; SCP: superficial capillary plexus; DCP: deep capillary plexus; OR: outer retina; ORCC: outer retinal chorio-capillaries; CC: chorio-capillaries; C: choroid; CT: choroidal thickness; SS: statistically significant

OCTA Parameters	Authors	Correlation	r and p-value
FAZ area	Zhang et al. [18]	Smaller FAZ in boys than girls	p=0.03
	İçel et al. [20]	SS negative correlation of FAZ area with CFT	p<0.0001
	Our study	SS negative correlation of FAZ area with CFT	r=-0.37, p<0.001
FAZ perimeter	Our study	SS negative correlation of FAZ perimeter with CFT	r=-0.37, p<0.001
FAZ circularity	Our study	Positive correlation of FAZ circularity with CFT	r=0.11, p=0.10
VD in SCP	Zhang et al. [18]	No significant correlation of VD in SCP with age & axial length	-
	Zhang et al. [18]	Higher foveal VD of SCP in boys than girls	p=0.03
	İçel et al. [20]	VD of SCP significantly reduced with increasing FAZ area	p=0.008
	Our study	VD of SCP had a significant negative correlation with FAZ area, FAZ perimeter and FAZ circularity	r=-0.28, p<0.001; r=-0.35, p<0.001; r=-0.14; p=0.03
	Our study	SS positive correlation of VD in SCP with CFT and SFCT	r=0.158, p=0.02; r=0.25, p<0.001
VD in DCP	İçel et al. [20]	Negative correlation of VD in DCP with age	p=0.015
	Zhang et al. [18]	No significant correlation of VD in DCP with age & axial length	-
	Zhang et al. [18]	Higher foveal VD in DCP in boys than girls	p=0.03
	İçel et al. [20]	VD in DCP significantly reduced with increasing FAZ area	p=0.004
	Our study	VD in DCP had a significant negative correlation with FAZ area and perimeter; and positive correlation with FAZ circularity	r=-0.25, p<0.001; r=-0.36, p<0.001; r=0.28, p<0.001
	Our study	SS positive correlation of VD in DCP with CFT and SFCT	r=0.11, p=0.09; r=0.20, p=0.004
VD in OR	Our study	VD in OR had a negative correlation with FAZ area and perimeter; and positive correlation with circularity	r=-0.13, p=0.06; r=-0.26, p<0.001; r=0.24, p<0.001
	Our study	SS positive correlation of VD in OR with CFT	r=0.14, p=0.04
VD in ORCC	Our study	VD in ORCC had significant negative correlation with BCVA (LogMAR)	r=-0.15, p=0.03
	Our study	VD in ORCC significantly correlated with SFCT	r=0.24, p<0.001
VD in CC	Al-Sheikh et al. [10]	VD in CC did not correlate with CT	-
	Abay et al. [23]	VD in CC significantly reduced with increasing age	-
	Our study	VD in CC significantly correlated with SFCT	r=0.23, p=0.001
VD in C	Maruko et al. [22]	VD in C significantly correlated with SFCT	r=0.738, p<0.01
	Our study	VD in CD significantly correlated with SFCT	r=0.22, p=0.001
CT	Al-Sheikh et al. [10]	CT reduced with increasing age and refractive error	r=-0.520, p<0.009; r=-0.402, p=0.025
	Our study	SS positive correlation of SFCT with VD in all layers except OR	Values given above

The OCTA parameters for the FAZ area and circularity measurements have a high repeatability [24]. However, the signal strength index values have been found to influence VD values and a higher SSI may lead to an increased or reduced VD value [25,26]. These findings underscore the importance of controlling SSI while using OCTA. In 40 eyes of 20 healthy adults having a mean age of 33.75±4.04 years, the mean VD of CC measured by Yun et al. was 101.44±3.48 with Plex Elite (Zeiss, Germany), 105.47±1.51 with DRI OCT-1 Atlantis (Topcon Medical Systems, Inc., Oakland, NJ), 94.67±11.29 with Zeiss AngioPlex, and 88.75±10.54 with Spectralis OCTA (Heidelberg Engineering, Germany) [27]. This shows that findings with different equipment may not be comparable. In our subjects falling in a comparable mean age, the mean VD of CC was 37.02±16.43 with SD-OCT (Nidek RS 3000 Advance 2). Faatz et al. recommended that results obtained using different devices being not similar should not be amalgamated in clinical trials [28]. We utilised SD-OCT for our study and we caution that the parameters given here may be of utility while studying subjects with the same or similar equipment.

The VD gets affected by age, blood pressure, and diurnal variation [27,29]. The scattering of light from the overlying retina and retinal pigment epithelium, difficulty in identifying each choroid capillary tube on account of tight arrangement and signal attenuation due to greater depth may also adversely affect the measurement of VD in CC and choroid using OCTA [10]. All these factors need to be considered while creating normative databases.

The potential limitation of our study was the use of 3 x 3 mm scans that do not include the arcades or the entire posterior pole. The current status of speed constraints and technology has disadvantage of a sparse scan density while capturing a wider scan. A less dense scan does not provide adequate pixel resolution to resolve deeper plexuses. We utilised narrower scans to maximise the resolution of deeper plexuses at the macula at the expense of a wider view [30].

## Conclusions

On quantitatively measuring FAZ parameters and VD of retino-choroidal layers, it was found that VD was the highest in outer retinal chorio-capillaries followed by choroid, chorio-capillaries, superficial capillary plexus and deep capillary plexus and was the lowest in the outer retina. Out of different layers, alteration in the VD of SCP, DCP and OR is more likely to affect FAZ parameters; an increase in VD causes reduction in the FAZ area/FAZ perimeter and an increase in FAZ circularity. In a healthy subject, a thicker CFT is associated with a higher VD in SCP, DCP and OR and a thicker SFCT implies a higher VD in almost all the layers except OR. Out of all the layers, only VD in ORCC significantly influenced BCVA, and lesser VD in this layer is likely to be accompanied by worse BCVA. The FAZ and retino-choroidal VD parameters can act as novel biomarker for diagnosing and giving prognosis of ocular diseases. Hence, values for the healthy population should be established for comparison.
